# The current status of prophylactic femoral intramedullary nailing for metastatic cancer

**DOI:** 10.3332/ecancer.2016.698

**Published:** 2016-12-01

**Authors:** NM Ormsby, WY Leong, W Wong, HE Hughes, V Swaminathan

**Affiliations:** 1 Orthopaedic Surgery, Wirral University Teaching Hospital NHS FT, Arrowe Park Road, Upton, Wirral CH49 5PE, UK; 2 University of Liverpool, L3 5TR, UK; 3 Palliative Care, Health Education East Midlands, NG11 6NJ, UK

**Keywords:** metastatic cancer, intramedullary nailing, arthroplasty, internal fixation, pathological fracture, prophylactic nailing

## Abstract

The most common site for cancer to spread is bone. At post-mortem, bony metastases have been found in 70% of patients dying from breast and prostate cancer. Due to the prevalence of cancer, bone metastasis and the associated management represents a huge burden on NHS resources.

In patients with metastasis, around 56% of these involve the lower limb long bones. Due to the huge forces placed upon long bones during weight bearing, there is a high risk of fracture through areas of metastasis. It is reported that 23% of pathological fractures occur in the femoral subtrochanteric region. This area is subjected to forces up to four times the body weight, resulting in poor union rate for these fractures, and significant morbidity associated with difficulty in mobilising, and in patient nursing.

As cancer treatments improve, the life expectancy in this subgroup of patients is likely to increase. Therefore medium-to-long-term management of these fractures, beyond the palliative, will become essential. We aim to evaluate the current management for metastatic malignant femoral disease, with particular focus on the prophylactic augmentation of diseased femorii using intramedullary nails.

## Introduction

Cancer currently accounts for one in every four deaths in the United Kingdom [1]. More than one-third of cancers are diagnosed in the over 1975s, and due to an ageing population this statistic is likely to increase [2]. The most common site for cancer spread is bone [3]. The most common cancers to metastasise to bone are breast and prostate. At post-mortem, bony metastases have been found in 70% of patients dying from breast and prostate cancer [4]. Other malignancies, such as thyroid, kidney, and bronchus, can also spread to bone, with a prevalence of around 30–40% in those who die from the primary malignancy [4].

Currently, metastasis to bone is associated with an adverse prognosis for these patients, increasing mortality [3]. Significant increased morbidity in these patients causes an increased burden on the health service. In patients with metastasis, around 56% of these involve the lower limb long bones [3]. Due to the huge forces placed upon long bones during weight bearing, there is a high risk of fracture through areas of metastasis. It is reported that 23% of pathological fractures occur in the femoral subtrochanteric region [5]. This area is subjected to forces of up to four times of body weight, resulting in poor union rate for these fractures, and significant morbidity associated with difficulty in mobilising, and in patient nursing. Symptoms commonly related to femoral metastatic disease include pain, difficulty weight bearing, and presence of a pathological fracture [6]. Pain from femoral metastasis is associated with subsequent mechanical instability that ultimately results in pathological fracture as the destruction of the bony cortex progresses.

As cancer treatments improve, the life expectancy in this subgroup of patients is likely to increase. Therefore, medium-to-long-term management of these fractures, beyond the palliative, will become essential. There are various surgical interventions that can be considered depending on the site and position of metastatic tumour and whether there is a fracture *in situ* or an impending fracture. The primary aim is to stabilise the femur, in order to maintain load-bearing ability and prevent subsequent fracture of the weakened cortices secondary to tumour infiltration. Instrumentation of the femur is reported to provide longitudinal structural support and pain relief, allowing the patient to weight bear through the limb [7, 8].

These factors are important as they relate to a patient's independence and ability to undertake normal daily activities. Given that some of these patients undergo prophylactic nailing as a palliative procedure, any improvement to quality of life can significantly affect their quality adjusted life years (QALYs). This principle is very important. The prophylactic management of bone metastasis may become an important feature of cancer management in the future. We aim to evaluate the current management for metastatic malignant femoral disease, with particular focus on the prophylactic augmentation of diseased femorii using intramedullary nails.

## Diagnosis

If a malignant lesion is strongly suspected in the femur, further investigations are essential to help confirm their presence, and identify the primary source. Grimer *et al*. recently published guidance in 2010 relating to the investigations that should be performed when a suspicious bony lesion is suspected [9].

In patients who have a pre-existing diagnosis of cancer, an assumption of metastatic disease must not be made without evidence to support this, as the management plan is vastly different. Bickels *et al*. have recommended a bone scan to exclude other lesions. If there are none found, then this should be managed like a primary tumour with biopsy to identify a diagnosis [7]. Any other painful sites should also be imaged with plain radiographs.

In patients with no previous history of cancer, a full systematic history and examination is crucial, combined with investigations. Depending on age, two different management pathways are suggested. Recommended imaging modalities include plain radiograph, magnetic resonance imaging (MRI), and isotope bone scan. Bone scans are required as above to identify if this is a solitary lesion or if there is extensive disease. Computed tomography (CT) scans are also crucial to help diagnose primary origin and stage of the disease. They can also be useful to quantify bony destruction and in surgical planning. A tissue diagnosis, however, is key in these patients, guiding the ultimate management of the patient, both from an oncological and from a orthopaedic point of view. If a primary lesion is identified, which is more amenable to biopsy, this is preferable, particularly if the bone is not amenable to surgery or surgery is not currently indicated.

## Biopsy of suspicious lesion

It is common practice in orthopaedics to send histology samples intraoperatively for patients with pathological lesions and fractures. In patients having arthroplasty surgery or modulated proximal femoral replacement, the excised portion of the femur is sent for suitable tissue analysis by a histopathologist. In those patients with more distal lesions who are being managed with intramedullary device, bone reamings are routinely sent to achieve a tissue diagnosis. A paper by Hassan found that tissue sent from reamings was inadequate in one third of patients, and a diagnosis from reamings alone was not possible for this group of patients [10]. This is due to the traumatic way the tissue is harvested, with significant heat generated by reaming. We should therefore not rely upon this for diagnosis. If at all possible a tissue diagnosis (from a core needle biopsy) would be beneficial prior to any intervention, surgical or otherwise. This enables an MDT decision to be made regarding the multiple important factors that will dictate treatment.

## Decision to treat with surgery

Several different tumour characteristics can influence the way a metastatic lesion is managed and must be considered prior to palliative operative management. It is vital to identify those tumours that respond predictably to oncological therapy without surgical intervention (such as lymphoma), and also primary musculoskeletal tumours (e.g. Sarcoma) that will require urgent specialist referral and intervention [7].

After ruling out the above, the next point is to determine the primary origin of the cancer. This will provide some insight into how aggressive the tumour will be, and how it will affect the bones. Will it cause lytic lesions or sclerotic? It can also be useful in the multi-disciplinary team (MDT) approach as some malignancies will respond better to chemo/radiotherapy and hormonal therapy than others. The origin of the tumour, if known, can also help with pre-operative optimisation. Some malignancies are known to be very vascular, such as bony metastases from a renal cell carcinoma (RCC). Chatziioannou *et al*. published literature on the significance of embolisation of RCC bone metastases prior to surgery. It was identified that there is a significant difference with blood loss during surgery (*p* = 0.049) and the need for subsequent blood transfusions (*p* = 0.03) when RCC metastases are completely devascularised compared to incomplete devascularisation [11]. These patients may require pre-operative devascularisation in the form of embolisations.

A patient’s estimated life expectancy can influence the decision to treat operatively. This is always difficult to assess and it is very individual to each patient. General consensus is that a survival of 6–12 weeks would be the minimum life expectancy required for relatively simple procedures to stabilise a bone such as IM nailing, and 6 months for complex reconstruction procedures [7]. Life expectancy varies greatly between tumour types. Other factors include, premorbid function, local disease control, and degree of spread. There have been many papers looking into positive and negative prognostic indicators in patients with bony metastasis. Pathological fracture, visceral metastasis, low haemoglobin ( < 7 mmol/L) and lung cancer were found to be negative predictors in one study, with myeloma positively prognostic [12]. A study by Nathan *et al*. found that independent negative predictors were performance status, number of bone metastases, visceral metastases, haemoglobin count. Interestingly, the most stastically significant prognostic value was the surgeon’s survival estimate itself [13].

## Isolated solitary lesion

Identification of patients with an isolated solitary metastasis warrants further more detailed assessment. The desire is often to fully excise localised solitary lesions, however, given that the disease is systemic by definition this is not curative. There is evidence showing that radical excision of solitary metastasis can provide a more prolonged disease-free interval and thus improving patient’s survivability [14]. This is particularly true for metastasis from renal cell carcinoma [15]. Baloch *et al*. reported in his case series of 25 patients one, three and five year cumulative survival rates of 88%, 54%, and 13%, respectively, following either, radical excision, amputations or with endoprosthetic replacement [14]. Hence, if patients demonstrated higher survivability outcome, resection with reconstruction may provide much more durable and reliable construct. Conversely, here is evidence that resection of lesions does not improve patient outcomes, but in general, patients with solitary lesions do better than those with multiple bony deposits [7].

## Pathological fractures

An untreated bone metastasis can lead to a pathological fracture. This is one of the most important factors affecting patient morbidity. The mechanism of how a tumour causes bony destruction is important to understand. There are three distinct phenotypes of bony metastasis: bony lysis, sclerotic lesions, and a mixed picture. This impacts the risk of pathological fracture. Osteolytic lesions (the classic being breast) stimulate osteoclastogenesis through tumour production of interleukin (IL)-1, IL-6, MIP1α, and RANK ligand. The tumour also stimulates existing osteoclasts through the production of tumour necrosis factor (TNF) and parathyroid hormone-related peptide, increasing RANK-L expression on osteoblasts [16]. Osteoblastic lesions (classically prostate) are the result of tumour cell-mediated activation of osteoblasts, through production of TGF-β and bone morphogenic protein [17]. Prostate tumour cells also demonstrate osteomimicry with the tumour cell differentiating towards an osteoblastic bone forming phenotype [18, 19]. Fractures will commonly occur through the area of metastatic lesions, especially in weight bearing bones. Features suggestive of impending pathological fracture include large lesions, lytic in nature, and erosion into or through a cortex of the femoral bone.

## Estimating the risk of pathological fracture

**Figure 1.**
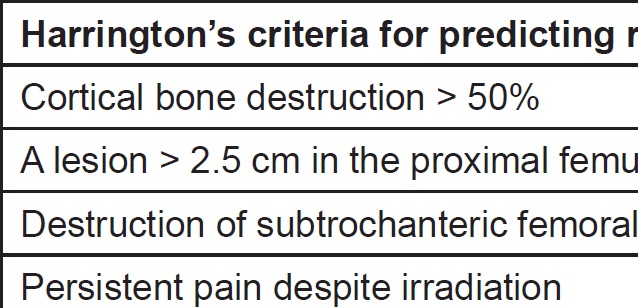
Harringtonʼs criteria for predicting risk of pathological fracture.

Once a metastatic lesion has been identified in the femur, tools are available to help quantify the risk of a pathological fracture. A scoring system for predicting this risk was originally developed by Harrington in 1986. He listed four criteria that could be used to predict the fracture risk of metastatic disease primarily in the femur (Figure 1). Previous studies state that the existence of one or more of the described criteria is thought to be an indication for prophylactic fixation of femur. However, limited statistical evidence is available to describe the sensitivity, specificity and reproducibility of these criteria [20–22].

**Figure 2.**
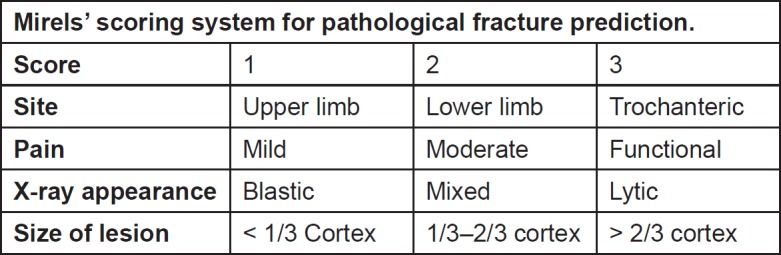
Mirelsʼ scoring system for pathological fracture prediction.

Mirels developed another scoring system in 1989, which is currently widely used to predict the risk of pathological fracture. This scoring system consists of four criteria, each scored from 1 to 3 (Figure 2). A score of ≤ 7 and ≥ 9 gives a fracture risk of 4% and 33%, respectively. He further recommended radiotherapy for patients with a score of ≤ 7, clinical judgement for a score of 8 and prophylactic fixation for patients with a score of ≥ 9. Several studies have validated Mirel’s scoring system, showing sensitivity and specificity to be 91% and 35%, respectively [23, 24].

There are limitations with the Mirels’ scoring; the low specificity and uncertainty with a score of 8 could lead to potentially unnecessary interventions being performed in cancer patients.

**Figure 3.**
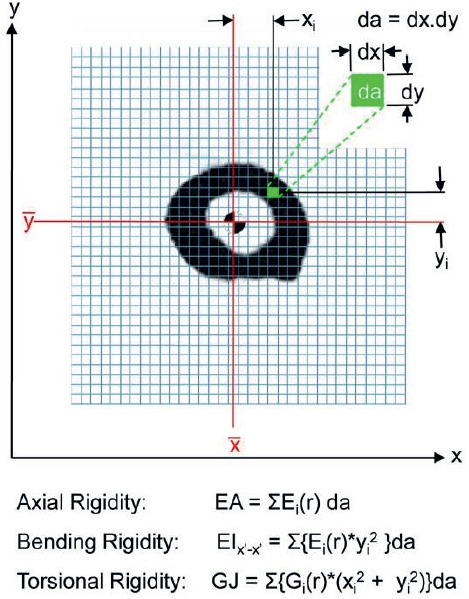
CT-based rigidity analysis (CTRA).

Recently, CT scan has been developed as an assessment tool for risk of impending fracture. Nazarian *et al* had developed and validated CT-based rigidity analysis (CTRA). CT scans of the bone involved and a hydroxyapatite phantom (CIRS Tissue Simulation and Phantom Technology) to standardise X-ray attenuation for each pixel to bone mineral density. The final calculation provides a measure of bone resistance to Uniaxial loads (EA), bending moment (EI) and torsional moment (GJ) (Figure 3).

The CTRA was found to have a sensitivity of 100% versus 67% and specificity of 60% versus 48% when comparing to Mirel scores (Mirel score ≥ 9). Folowing initial consultation, patients who were deemed to have high risk of impending fractures received stabilisation and were excluded from Damron *et al*. study. Hence, the study can only be generalised to patient population whereby the benefit of prophylactic stabilisation was questionable following initial assessment.

Despite the clear superiority in sensitivity and specificity, it is worth pointing out that CTRA is not as easily accessible in a clinical setting. In some cases, it will be quite apparent from clinical assessment and Mirel scoring regarding the impending risk of fracture. In cases whereby Mirel scoring were inconclusive, CTRA can be performed to provide further information which might guide treatment [25–26].

Tatar *et al*. also examined the correlation of size of long bone metastasis with percentage of cortical bone involvement measured on CT scan to risk of fractures in 47 patients who were treated with radiotherapy. They have concluded that the only positive predictive parameter for fracture was circumferential cortical involvement of ≥30% with recommendation of nailing for any patients within this group in their Level IV study [27].

## Indications for surgical management

The major indications for surgical management of femoral metastasis are in patients with impending or pathological fractures, and in those with intractable pain [28–30]. Several options are available for surgical management. Prophylactic internal fixation of the femur for metastatic disease was first described by Griessman *et al* in 1947. Since then, a number of case reports and studies have been published with intramedullary nailing becoming the treatment of choice for impending femoral fractures caused by metastases [31]. A recent study showed that patients who underwent prophylactic fixation have improved post-operative outcomes than patients who underwent fixation after pathological fracture, including longer survival, and shorter hospital stay [32].

## Nailing

Despite a long history of performing intramedullary nailing, limited evidence exists which evaluates the most appropriate surgical implantation technique. Historically, cephalomedullary nails were used for prophylactic fixation as it provides greater stability for the entire femur including the femoral neck as opposed to plating. However, conflicting evidence has been identified in different studies and it remains uncertain if femoral neck fixation is necessary in patients with subtrochanteric metastatic lesions. Study by Moon *et al* found no femoral neck lesions in follow-up of 145 nailings in 141 patients with subtrochanteric metastatic lesion. Hence, they do not recommend stabilising the femoral neck in these group of patients [33]. Alvi and Damron found incidence of disease progression was considerably lower than the complication rate potentially attributable to the use of cephalomedullary implants, but this is very difficult to say categorically. Only one patient in this series developed a new lesion not previously recognised, but 12% progressed [34]. Fixation with nails is better than plates and screws, although not periprosthetic [32]. Biomechanically speaking a reamed nail does provide a more stable construct in comparison to undreamed nail. However, Cole *et al*., in their paper suggested that there is evidence that reaming is no better, and that reamings are not ideal diagnostically. Hence, undreamed nails would be best, reducing trauma and operative time [35].

Venting is a method developed to reduce the intramedullary pressure during nail insertion. It has been hypothesised that a raised intramedullary pressure could lead to some surgical complications. A study showed that venting could reduce intramedullary pressure in a femur with metastatic disease, but not low enough to prevent fat and tumour embolisations. It is also unclear how venting affects the spreading of cancer locally and systematically [36]. A questionnaire has been developed for orthopaedic surgeons in Canada which asked if they routinely use venting when treating impending femoral fractures. The results showed that there is no general consensus regarding the routine use of venting [37].

## Complications of intramedullary nailing

Several complications are associated with prophylactic fixation of metastatic femoral disease. These include general surgical risks such as infection (1–2%), deep vein thrombosis (4.9–14%), and intraoperative (1%) or postoperative death (3 months mortality rate of roughly 13%, 1-year mortality rate of 70–90% depending on cancer type) with mean post-operative survival time of 14 months [32, 38, 39]. Procedure-specific risks include fat embolisation, failure of implant (2–8%), seeding of cancer, and periprosthetic fracture. Overall, complication rate related to surgery is approximately 8% [32, 38, 39]. There remains uncertainty with regard to the effects of intramedullary nailing on local and systematic spreading of cancer with some studies indicating that there are no significant effects, while others identified some evidence of the tumour spreading [31].

## Arthroplasty

As the proximal femur is one of the commonest sites of bone metastases, arthroplasty has a role in managing impending proximal femur fractures. Arthroplasties are often performed when there is acetabular involvement or presence of severe bony destruction and when any attempts of internal fixation are likely to be frutile. Surgical resection of the tumours followed by endoprosthetic reconstruction is a proven treatment option and allows for immediate weight bearing and reducing risks of construct failure post-surgery. However, these procedures are very complicated and should only be performed in specialist orthopaedic centres [38, 40–42].

## Plating

When the distal femur is affected, plating could be used as an option to stabilise the affected part of the femur. Generally, intramedullary nails are the devices of choice for fixation as they offer more extensive stabilisation of bone, a reduced risk of future fracture and a lower rate of fixation failure as opposed to plating. We were, however, unable to find any articles comparing the prophylatic use of intramedullary nailing and plating. Plating is generally reserved for pathological fractures where the use of intramedullary devices are contraindicated, such as the presence of densely sclerotic lesions or when an unstable metaphyseal fragments that could not be stabilised with intramedullary construct are present [43].

## Non-operative treatment

Metastatic bone disease is complicated and treatment requires a MDT approach to provide patients with the best outcome. Depending on the size of tumour and the patient’s comorbidities, metastatic femoral disease could primarily or initially be managed medically. Treatment options include chemotherapy, radiotherapy, bisphosphonates and hormonal therapy. Bisphosphonates have been found to reduce bone pain and delay the development of complications to bone [44]. No coherent data between studies exists on fracture risks following medical management of metastatic femoral disease; some papers suggested roughly 5–10% risk of fracture [32, 38]. Others show no difference between surgically or medically treated patients and some favour surgical management [45]. It is however noted that all these papers have a relatively small sample size and results published may have low statistical power.

## Fitness for surgery

Regardless of surgical techniques available, a patient must ultimately be a candidate for surgery. Patient selection is always an important influencing factor, and is more complicated particularly in patients with metastatic disease. The assessment for suitability for surgical measures also becomes increasingly important in the elderly population, given their associated health confounding comorbidities; age alone however is not an accurate independent predictor of surgical outcome [46]. Often it is these comorbidities or perceived fitness status that declares a patient unsuitable for surgical management [47]. The concept of frailty further confounds the decision to proceed to surgery, especially in an older patient. A patient’s current status with regards to daily activities, mobility and functioning are all highly relevant to prophylactic surgery of the femur. An evaluation needs to be made to ascertain the potential benefit gained from surgery. Previous functional status prior to metastasis to the affected limb, alongside prognostic values for the primary disease are required information. A multidisciplinary approach is therefore a must for these patients.

## Conclusions

Changing demographics and an ageing population forecast an increased incidence in cancer and consequential secondary complications such as bone metastases. There is therefore an essential growing need to establish which management pathway and method of prophylaxis for pathological fracture is the most effective in improving prognosis. Inevitably, establishing a universal solution is difficult solely due to the complexity of cancer treatment. Treatment is dependent on each particular patient and their bespoke health needs and fitness.

With criteria available to help recognise patients at high risk from femoral shaft fracture, it is important to have practical preventative interventions in place. These treatments will undoubtedly become increasingly relevant in the future of orthopaedics. Prophylactic fixation aims to stabilise the affected area and therefore prevent pathological fractures, in addition to improving mobility, patient prognosis and quality of life. There is reason to suggest that intramedullary nails could be used for effective prophylaxis, however the current lack of evidence surrounding surgical interventions is not sufficient to concretely justify which method has the most potential. The authors propose to create a pragmatic algorithm for the diagnosing clinican for the management of these fractures, and plan to report these findings upon completion.
